# A novel intragenic deletion in *OPHN1* in a Japanese patient with Dandy-Walker malformation

**DOI:** 10.1038/s41439-018-0032-8

**Published:** 2018-12-05

**Authors:** Aritoshi Iida, Eri Takeshita, Shunichi Kosugi, Yoichiro Kamatani, Yukihide Momozawa, Michiaki Kubo, Eiji Nakagawa, Kenji Kurosawa, Ken Inoue, Yu-ichi Goto

**Affiliations:** 1 Department of Clinical Genome Analysis, Medical Genome Center, National Center of Neurology and Psychiatry (NCNP), Kodaira, Tokyo, 187-8551 Japan; 2 Department of Child Neurology, National Center Hospital, NCNP, Kodaira, Tokyo, 187-8551 Japan; 3 Laboratory for Statistical Analysis, RIKEN Center for Integrative Medical Sciences, Yokohama, 230-0045 Japan; 4 Kyoto-McGill International Collaborative School in Genomic Medicine, Kyoto University Graduate School of Medicine, Kyoto, 606-8507 Japan; 5 Laboratory for Genotyping development, RIKEN Center for Integrative Medical Sciences, Yokohama, 230-0045 Japan; 6 RIKEN Center for Integrative Medical Sciences, Yokohama, 230-0045 Japan; 7 Division of Medical Genetics, Kanagawa Children’s Medical Center, Yokohama, Kanagawa 232-8555 Japan; 8 Department of Mental Retardation and Birth Defect Research, National Institute of Neuroscience, NCNP, Kodaira, Tokyo, 187-8551 Japan; 9 Department of Bioresource, Medical Genome Center, NCNP, Kodaira, Tokyo, 187-8551 Japan

**Keywords:** Mutation, Neurodevelopmental disorders

## Abstract

Dandy-Walker malformation (DWM) is a rare congenital malformation defined by hypoplasia of the cerebellar vermis and cystic dilatation of the fourth ventricle. Oligophrenin-1 is mutated in X-linked intellectual disability with or without cerebellar hypoplasia. Here, we report a Japanese DWM patient carrying a novel intragenic 13.5-kb deletion in *OPHN1* ranging from exon 11–15. This is the first report of an *OPHN1* deletion in a Japanese patient with DWM.

Dandy-Walker malformation (DWM) is a midbrain–hindbrain malformation characterized by cerebellar vermis hypoplasia and dysplasia, cystic dilatation of the fourth ventricle and an elevated torcula, often accompanied by hydrocephalus^1^. The frequency of DWM in the U.S. is ~1 in 25,000–35,000 liveborn infants (https://rarediseases.org/rare-diseases/dandy-walker-malformation/). DWM becomes apparent in early infancy, is complicated by macrocephaly, and occurs along with increased intracranial pressure, spastic paraparesis, and hypotonia^2^. In addition, motor deficits, such as delayed motor development, hypotonia, and ataxia, as well as intellectual disability (ID), are often seen^1,2^. To date, various chromosomal abnormalities, such as trisomy 9, −13, −18 and partial duplications/deletions of chromosomes, in DWM patients have been reviewed^1^. Additionally, heterozygous deletions of cerebellum-specific Zinc-finger genes, *ZIC1* and *ZIC4*, on chromosome 3q24 are associated with DWM^3^. X-linked DWM with ID is also caused by an *AP1S2* mutation^4^.

*OPHN1* encodes oligophrenin 1, which is a Rho-GTPase activating protein involved in synaptic morphogenesis and functions through the regulation of the G protein cycle^5^. *OPHN1* (NM_002547) consists of 25 exons and spans ~391 kb on chromosome Xq12 (UCSC Genome Browser: https://genome.ucsc.edu). Oligophrein 1 is an 802 amino-acid protein harboring multiple domains, such as a BAR domain, PH domain, Rho-GAP domain, and three proline-rich sequences^6^. *OPHN1* was originally identified as a disrupted gene by a translocation t(X;12) in a female patient with mild mental retardation^7^. To date, 10 point mutations, four splicing mutations, six small insertion/deletion or duplication mutations, and 17 chromosomal rearrangements in *OPHN1* have been identified in patients with neurodevelopmental disorders, including cerebellar hypoplasia, intellectual disability (ID), epilepsy, seizure, ataxia and schizophrenia (Human Gene Mutation Database, Professional 2018.2). In addition, Schwartz et al.^6^ recently expanded the clinical spectrum of OPHN1-associated phenotypes in comparison to the phenotypes described in previous reports. Moortgat et al.^8^ also described four families with intellectual disability without cerebellar hypoplasia.

Here, we report a Japanese DWM boy carrying an intragenic deletion in *OPHN1*.

The biobank at the National Center of Neurology and Psychiatry (NCNP) is a unique biorepository for neuropsychiatric, muscular, and developmental diseases in Japan (https://ncbiobank.org/index-e.html). We collected DNA samples, clinical information, and cell lines, with informed consent, from 583 families with neurodevelopmental diseases that were diagnosed between 2004 and 2016. The study was approved by the ethical committee of NCNP. The cell lines were developed by the immortalization of peripheral lymphocytes with Epstein-Barr virus.

We conducted the candidate gene approach on chromosome X to identify the causative gene in this patient. The following 19 known causative genes for XLID were analyzed by repeat expansion analyses (*FMR1* and *FMR2*) and Sanger sequencing (*PQBP1, ARX, MECP2, ATRX, RPS6KA3, IL1RAPL1, TM4SF2, PAK3, FACL4, OPHN1, AGTR2, ARHGEF6, GDI1, SLC6A8, FTSJ1, ZNF41*, and *DLG3*). We performed direct sequencing of PCR amplicons using an ABI3730 capillary sequencer (Thermo Fisher Scientific, Waltham, MA, USA) according to the standard protocol. We determined the breakpoint by comparing the sequence of the patient with sequences from an unaffected person.

The patient (III-1) was a 2-year-old boy, and he was referred for developmental delay at the age of 11 months. His maternal uncle was affected with hydrocephalus (Fig. 1a). The boy was born through normal delivery without asphyxia at 39 weeks of gestation. His weight and head circumference were 2756 g (−0.6 SD) and 32 cm (−0.9 SD), respectively. He acquired head control at 8 months, but he could not sit alone or speak words. Brain computed tomography (CT) and brain magnetic resonance imaging (MRI) suggested DWM (Fig. 1b). When he was 1 year old, he received shunting for his hydrocephalus. At the age of 1 year and 3 months, his height, weight, and head circumference were 81 cm (+1.1SD), 10.7 kg (+0.8SD), and 47 cm (0SD), respectively, and his developmental quotient (DQ) was 57.

**Fig. 1:**
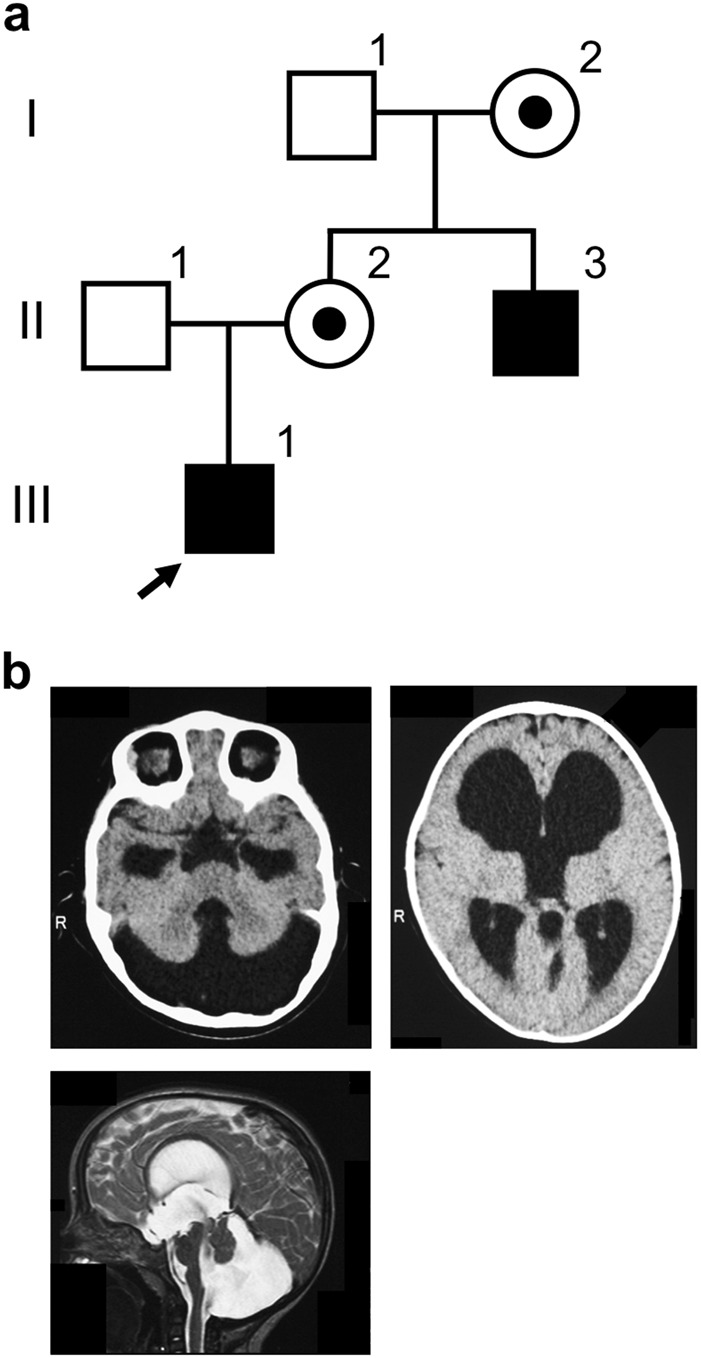
The pedigree and the brain images. **a** Pedigree of the family. Arrow indicates the proband. **b** Brain computed tomography images (Upper) and a T2-weighted magnetic resonance image (Lower) of the patient. Prominent cerebellar hypoplasia, an enlarged fourth ventricle and posterior fossa, and ventriculomegaly were noted

After initial screening for an *L1CAM* mutation by sequencing and a gross copy number variation in chromosome X by a BAC-based array-CGH^9^, which were both negative, we performed mutation screening of 19 known causative genes for XLID in the patient. Consequently, we found an intragenic deletion in *OPHN1* involving exon 11–15, which include the PH and GAP domains (Fig. 2a). To more precisely determine the mechanism of the deletion, we performed deletion mapping by PCR-based sequence-tagged site content mapping, followed by direct sequencing of the junction fragment. We narrowed the breakpoint region to a 437-bp PCR product amplified by a set of PCR primers derived from intron 10 and intron 15 (Supplementary Fig. 1). Direct sequencing of the PCR product containing the recombination breakpoint revealed that the deletion only occurred within a common five-nucleotide motif (AATTA) in intron 10 and intron 15, both in the patient and his mother. No low copy repeats or segmental duplications were found adjacent to the deletion breakpoints, suggesting that the genomic rearrangement occurred by a microhomology-mediated mechanism and not by non-allelic homologous recombination (Fig. 2b). The deletion spanned from 4218 nucleotides downstream of the exon 10-donor site (c.933 + 4,218) to 4081 nucleotides downstream of exon 15 (c.1276 + 4,081). The size of the deletion was 13,517 bp in length (GRch37/hg19: chrX:67,408,680-67,422,196). This deletion was also absent in three public databases, dbVar, ClinVar and the Database of Genomic Variants (http://dgv.tcag.ca/dgv/app/home). In addition, we did not find the deletion in a 1254 Japanese general population data set created by high-depth whole genome sequencing^10^.

**Fig. 2:**
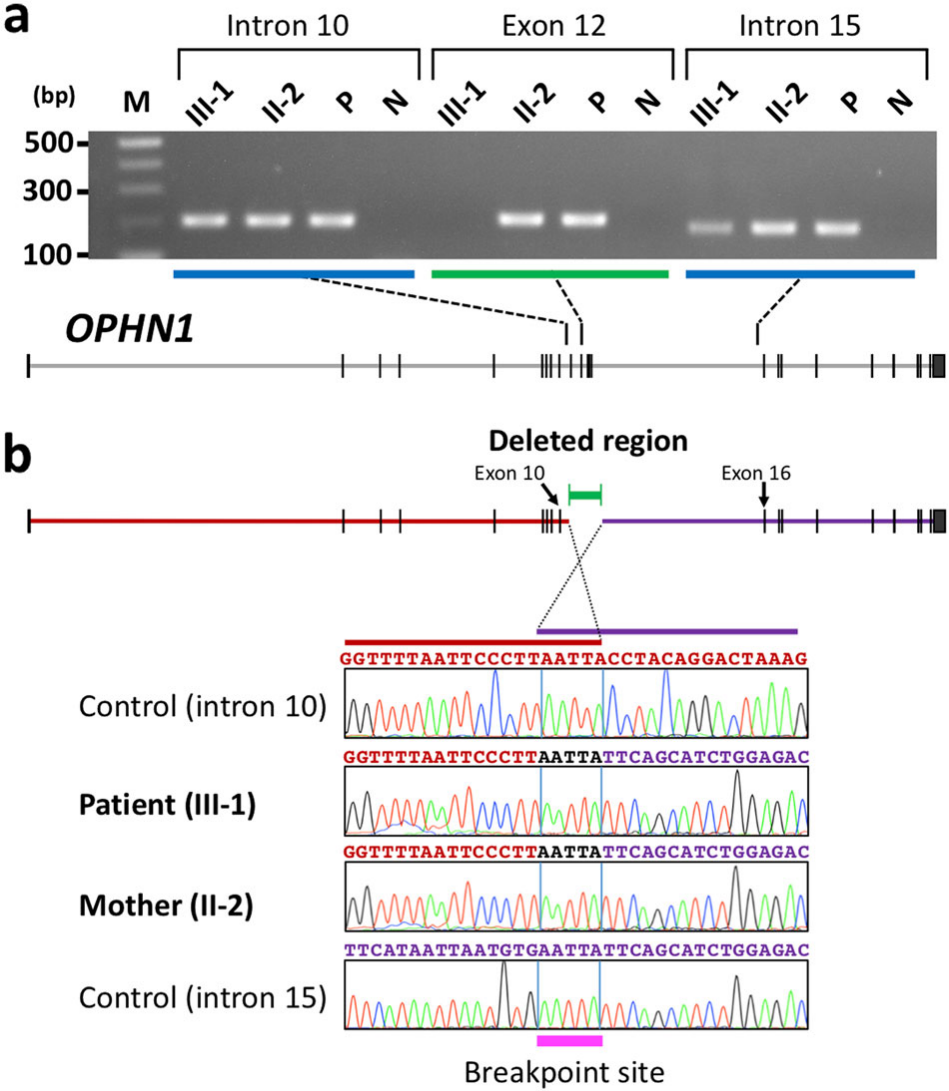
**Identification of an intragenic deletion in** ***OPHN1*** **in the patient III-1.** **a** Agarose gel image of the PCR products corresponding to intron 10, exon 12, and intron 15 in *OPHN1* in the patient and his mother (II-2). Exon 12 in the patient was deleted. The genomic structure of *OPHN1* is also shown in the agarose gel image of each PCR analysis. P positive control DNA from an unaffected person, N negative control (H_2_O), M 100-bp ladder DNA size marker. **b** (Upper) the deletion map of *OPHN1*. The green rectangle with end bars shows the deleted region. The deletion encompasses 13.5 kb from intron 10 to intron 15. (Lower) The region proximal to the deletion, shown as a red bar, and the region distal to the deletion, shown as a blue bar. Sequence chromatograms of the junction fragment containing the breakpoint site from the patient and his mother are shown in the middle two rows. An overlap of a five-nucleotide motif (AATTA) is shown as a pink bar. Reference sequences of the corresponding regions in intron 10 (upper) and intron 15 (lower) are shown

In past studies, *OPHN1* mutations have been reported in cases with XLID with cerebellar hypoplasia, strabismus, epilepsy, hypotonia, ventriculomegaly, and distinctive facial features^6,8^. Additionally, *OPHN1* mutations have also been reported in individuals with autism or childhood onset schizophrenia^11^, so *OPHN1*-associated clinical phenotypes are variable^6–8,11^. DWM and hydrocephalus in the present patient are likely the most severe imaging findings observed in patients with *OPHN1* mutations. The novel intragenic deletion in *OPHN1* eliminated exon 11–15, which encode PH and GAP domains. This deletion leads to a premature truncation, c.934_1276del (p.Gly312Ilefs*24), of *OPHN1*; the transcript might presumably be degraded by nonsense-mediated mRNA decay. Altogether, we concluded that the deletion in *OPHN1* is the pathogenic genetic abnormality in this patient who showed profound ID and DWM.

## Electronic supplementary material

Supplementary Figure 1

## Data Availability

The relevant data from this Data Report are hosted at the Human Genome Variation Database at 10.6084/m9.figshare.hgv.2405
