# Supplementary figures and images for: A novel intragenic deletion in OPHN1 in a Japanese patient with Dandy-Walker malformation
Source: Hum Genome Var. 2018 Dec 5;6:1. doi: 10.1038/s41439-018-0032-8 (PMC6281661; doi:10.1038/s41439-018-0032-8)

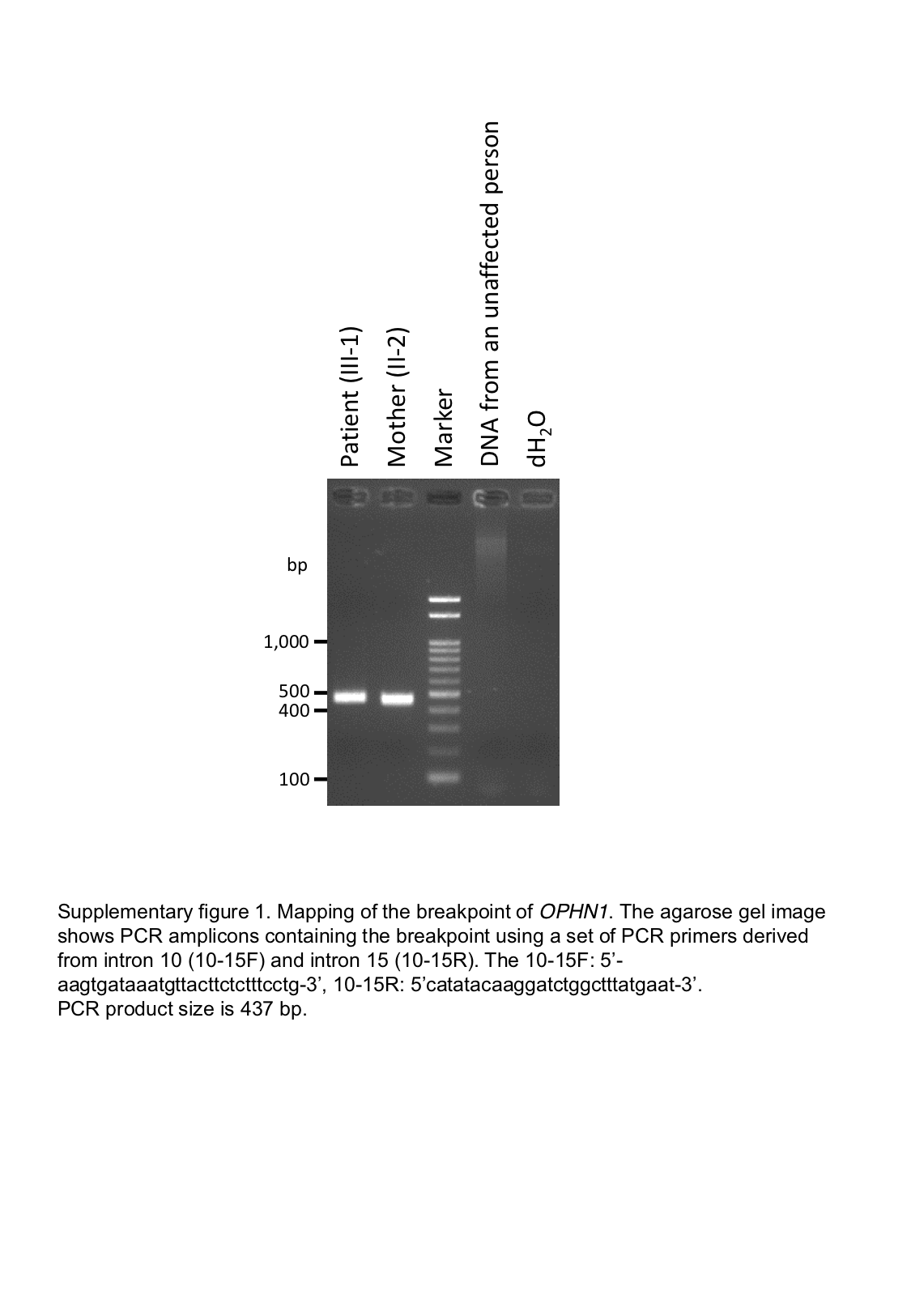

Supplement: Supplementary file 1 — Supplementary Figure 1 [file 41439_2018_32_MOESM1_ESM.tif]
